# Hereditary Character of Cancer

**Published:** 1874-09

**Authors:** 


THE HEREDITARY CHARACTER OF CANCER.—* * * The following recently discovered letter from Dr. Short, who was the chief medical officer at St. Helena at the time of Napoleon's death, will perhaps be of interest:

"St. Helena, May 7th, 1821.—You will doubtless be very much astonished to learn that Napoleon died on the 5th of May, after a prolonged illness. His disease was cancer of the stomach, which undoubtedly began to develop already many years ago, though it was only during the past few months that it took on a condition of ulceration. A few days before his death I was frequently called to take part in the consultations which were held, but never saw him professionally—owing to his unwillingness to see strangers—until just before his death. The face of the dying man was the most beautiful I ever saw; his features were characterized by the most perfect peacefulness and gentleness. The following day I was present at the post-mortem examination, which was made at Napoleon's own special request, for the purpose, he said, that his son might be put upon his guard, in case the disease proved hereditary in its nature. During his entire illness he had not shown the slightest disposition to complain, but maintained to the very end his wonderful mastery over himself. It appears that Napoleon's father died of the same disease, and his sister, the Princess Borghese, was also supposed to have died of it. Hence climate and mode of life could have had nothing to do with causing his death."—*Deutsche Versicherungs-Zeitung.*

